# Intravenous thrombolysis in acute ischemic stroke after reversal of warfarin anticoagulation in patients with mechanical heart valve prostheses and an INR > 1.7

**DOI:** 10.3389/fneur.2026.1937344

**Published:** 2026-10-07

**Authors:** Jiri Neumann, David Cernik, Jan Bilik

**Affiliations:** 1 Stroke Center, Department of Neurology, Chomutov Hospital, Krajska zdravotni as, Chomutov, Czechia; 2 Comprehensive Stroke Center, Department of Neurology, Palacký University Medical School and University Hospital Olomouc, Olomouc, Czechia; 3 Comprehensive Stroke Center, Department of Neurology, Masaryk Hospital, Krajska zdravotni as, Ústí nad Labem, Czechia; 4 Emergency Medical Service of the Usti nad Labem Region, Ústí nad Labem, Czechia

**Keywords:** acute ischemic stroke, four-factor prothrombin complex concentrate, intravenous thrombolysis (IVT), mechanical heart valve, warfarin

## Abstract

**Background:**

Acute ischemic stroke (IS) frequently occurs in patients receiving oral anticoagulant (OAC) therapy. Although warfarin has largely been replaced by direct oral anticoagulants in routine clinical practice, it remains the only approved anticoagulant for certain indications, particularly in patients with mechanical heart valve protstheses. In these patients, the international normalized ratio (INR) is the key determinant of eligibility for intravenous thrombolysis (IVT). We present a potentially effective therapeutic strategy involving rapid reversal of warfarin anticoagulation with four-factor prothrombin complex concentrate (4F-PCC) followed by IVT.

**Methods:**

We report two patients with similar clinical presentations who were receiving long-term warfarin therapy because of a mechanical aortic valve replacement. 76-year-old man with AIS had complete paralysis of the right lower limb, paresis of the right upper limb, and right central facial palsy. INR was 3.2. The second patient was a 76-year-old woman with dysarthria, left central facial palsy and left-sided hemiparesis. Her admission INR was 1.91. In both patients, CT angiography did not demonstrate arterial occlusion suitable for endovascular therapy.

**Results:**

In both patients, four-factor prothrombin complex concentrate (4F-PCC) was administered to achieve rapid normalization of the INR. This was immediately followed by standard IVT. Both patients experienced marked neurological improvement, with no hemorrhagic or thromboembolic complications. At the 3-month follow-up, both had achieved complete functional recovery without residual neurological deficits. In conclusion, it can be stated that the chosen procedure led to an excellent clinical outcome in accordance with previously published cases, in the absence of other standard options for recanalization therapy.

## Introduction

Ischemic stroke (IS) frequently occurs despite ongoing oral anticoagulant (OAC) therapy, particularly when prescribed for the prevention of cardioembolic events in patients with non-valvular atrial fibrillation (AF), following mechanical heart valve replacement, or for the treatment and secondary prevention of venous thromboembolism. Oral anticoagulants comprise vitamin K antagonists (VKAs), which inhibit the synthesis of vitamin K-dependent coagulation factors, and direct oral anticoagulants (DOACs), which directly inhibit either thrombin (dabigatran etexilate) or activated factor Xa (apixaban, edoxaban, and rivaroxaban). Owing to their more favorable pharmacological and safety profiles, DOACs have largely replaced VKAs in routine clinical practice over the past decade (1). Nevertheless, VKAs remain an important therapeutic option for selected patient populations. One clinical setting in which VKAs remain indispensable is anticoagulation in patients with mechanical prosthetic heart valves. In the Czech Republic, warfarin is currently the only available VKA.

Despite appropriate OAC therapy for the prevention of cardioembolic stroke, patients remain at risk of acute ischemic stroke. Recent clinical studies and meta-analyses have reported an annual incidence ranging from 1 to 9% among patients receiving OAC therapy, although the incidence may be even higher in routine clinical practice (2–6). Li et al. reported an annual IS risk of 2.6–5.0% in patients treated with VKAs, with the risk strongly associated with the proportion of time spent within the therapeutic international normalized ratio (INR) range (7). In a large multicenter cohort of 38,632 patients with acute IS admitted to 23 Swiss stroke centers, 2,368 patients (6.1%) were receiving VKA therapy at stroke onset (8). Among observational studies evaluating acute IS in patients treated with VKAs, one included 586 patients (8.2%) with mechanical heart valve prostheses among a total cohort of 7,176 patients with AF (9).

In patients taking VKAs, IVT is indicated only if the INR is ≤1.7. Current guidelines consider an INR > 1.7 a contraindication to IVT due to concerns about an increased risk of symptomatic intracranial hemorrhage (SICH) (10–12). Furthermore, prior reversal of the VKA effect with prothrombin complex concentrate to enable subsequent IVT is not routinely recommended because there are insufficient data regarding the safety and efficacy of this approach, and concerns remain regarding potential thromboembolic complications. In patients with a mechanical heart valve prosthesis, the specific risk of prosthetic valve thrombosis associated with reversal of anticoagulation must also be taken into account.

Here, we present two cases of acute ischemic stroke in which urgent reversal of warfarin anticoagulation with four-factor prothrombin complex concentrate (4F-PCC) was followed by successful intravenous thrombolysis in patients presenting with an INR > 1.7.

## Methods

Cases from two centers were retrospectively analyzed. Inclusion criteria were the presence of clinically significant ischemic stroke, the presence of a mechanical aortic valve, warfarin use and an initial INR above 1.7, administration of 4F-PCC and IVT. Initial examination included standard CT and CTA. Contraindications to intravenous treatment were standard except for the use of anticoagulant therapy and the presence of a therapeutic INR. Clinical status was assessed according to the National Institutes of Health Stroke Scale (NIHSS) and modified Rankin Scale (mRS) at discharge and at a three-month interval. The safety profile was assessed by the presence of any hemorrhagic or thrombotic complications in the first 7 days after treatment.

## Results

Only two patients were included in two centers, given the relatively rare occurrence of these cases. A 76-year-old man was transported by air emergency medical services directly to our comprehensive stroke center after a positive FAST PLUS test from a location outside the catchment area of the nearest primary stroke center. On admission, neurological examination revealed a right central facial palsy, severe right-sided hemiparesis, and tactile hypoesthesia of the right extremities. His NIHSS score was 9.

The patient had been receiving warfarin therapy since undergoing mechanical aortic valve replacement in 2015. His medical history also included arterial hypertension, hyperlipidemia, and type 2 diabetes mellitus.

Point-of-care INR testing demonstrated a therapeutic INR of 3.2. Non-contrast brain computed tomography (CT) showed no acute abnormalities, and CT angiography of the extracranial and intracranial cerebral arteries revealed neither large-vessel occlusion nor significant arterial stenosis.

Given the severity of the neurological deficit, intravenous thrombolysis (IVT) was considered appropriate after careful assessment of the potential risks and benefits and following informed patient consent. As the first step, four-factor prothrombin complex concentrate (4F-PCC; Ocplex®) was administered at a dose of 30 IU/kg, determined according to the baseline INR of 3.2. Repeat INR measurement immediately after reversal was 1.2. Intravenous alteplase (Actilyse®) was subsequently administered at a total dose of 65 mg according to the standard protocol, consisting of an initial 10% intravenous bolus followed by a 60-min infusion.

The door-to-needle time was 37 min. This delay was attributable not only to warfarin reversal but also to an unexpected CT scanner malfunction requiring transfer of the patient to a backup scanner. The onset-to-treatment time was 98 min. Vitamin K was intentionally withheld because of the presence of a mechanical aortic valve prosthesis. Serial INR measurements after IVT remained stable and did not exceed 1.6.

Follow-up brain CT demonstrated no evidence of intracranial hemorrhage. CT was performed just 19 h after IVT, taking into account the risk of thrombotic complications associated with the mechanical valve. Bridging therapy with LMWH was administered while warfarin therapy was being re-established. Brain magnetic resonance imaging subsequently confirmed a cortical infarction in the left frontal lobe (Figure 1). The patient was transferred to his regional stroke center for continued management and inpatient rehabilitation.

**Figure 1:**
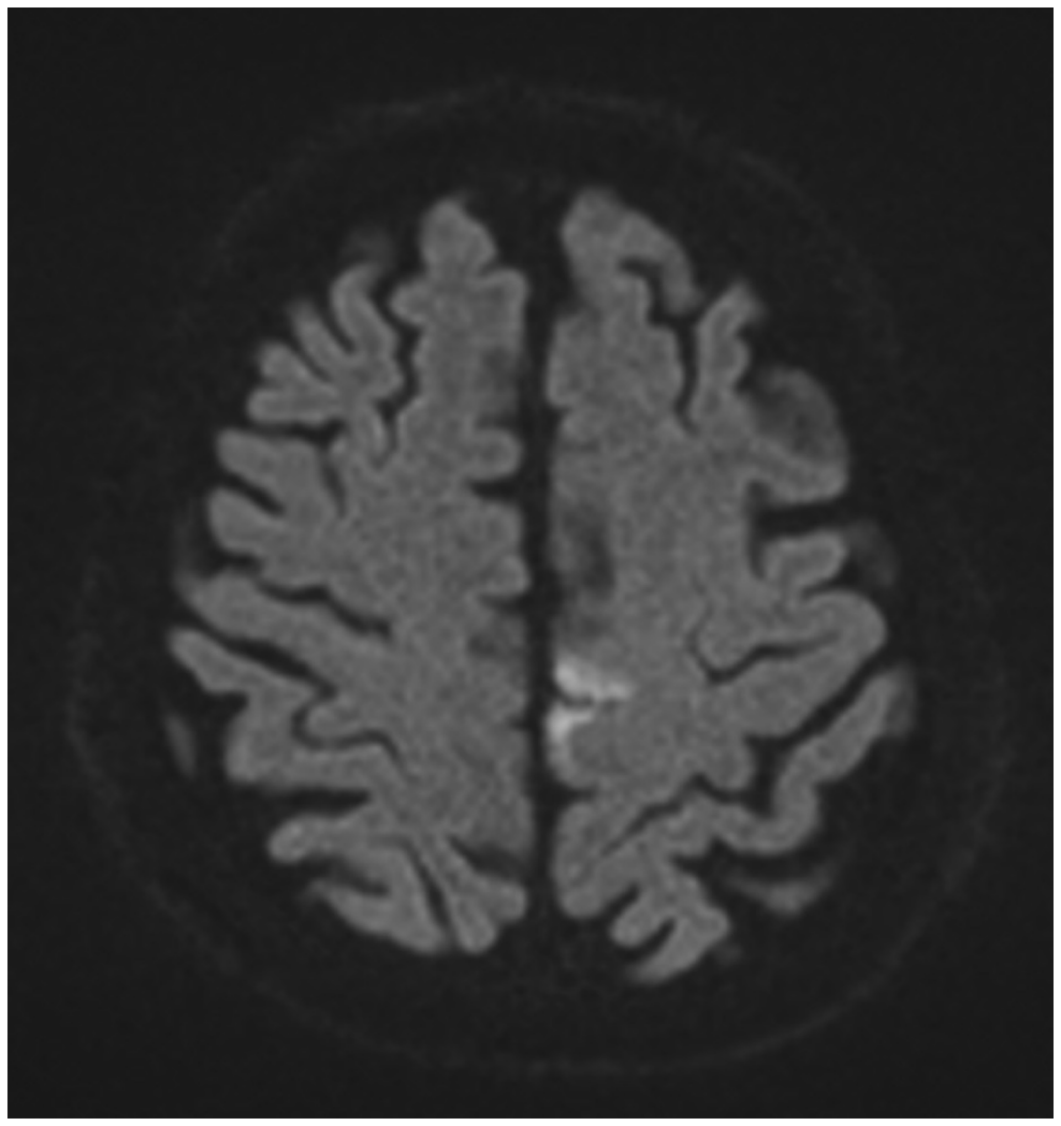
Ischemic changes in the left frontal lobe on diffusion-weighted imaging MR.

His neurological status improved to mild right-sided hemiparesis, with further recovery during rehabilitation. He was discharged home on day 12, independently ambulatory and fully independent in activities of daily living, with a mRS of 2. INR at discharge was 2.83. At the 3-month follow-up, he had achieved an excellent functional outcome, with a mRS of 0.

A 76-year-old woman presented to our stroke center (CT unit) approximately 1.5 h after symptom onset with dysarthria, left central facial palsy, left-sided hemiparesis, and tactile hypoesthesia of the left extremities. Her National Institutes of Health Stroke Scale (NIHSS) score fluctuated between 7 and 9.

The patient had been receiving long-term warfarin therapy (3 mg daily) following mechanical aortic valve replacement. Her medical history was also significant for arterial hypertension and hyperuricemia.

Non-contrast brain computed tomography (CT) demonstrated no acute abnormalities, while CT angiography revealed neither intracranial nor extracranial large-vessel occlusion or significant arterial stenosis. Point-of-care INR testing using a bedside coagulometer showed an INR of 1.91.

Four-factor prothrombin complex concentrate (4F-PCC; Ocplex®) was administered at a dose of 1,500 IU (25 IU/kg for a body weight of 60 kg). Immediately after completion of the infusion, repeat INR was 1.3, and intravenous alteplase (Actilyse®) was initiated without delay according to the standard dosing protocol at a total dose of 72 mg. The door-to-needle time was 31 min. Vitamin K was intentionally withheld.

The patient showed marked neurological improvement within the first 24 h after treatment. Follow-up non-contrast brain CT demonstrated neither cerebral infarction nor intracranial hemorrhage. No other complications related to either 4F-PCC administration or alteplase treatment were observed. Serial INR measurements following IVT remained stable, ranging between 1.4 and 1.7. In this case, too, the CT scan was performed earlier, 18 h after IVT, and bridging therapy with LMWH was initiated until a therapeutic INR was achieved.

The neurological deficit continued to improve during targeted rehabilitation. Warfarin therapy was resumed at a slightly increased maintenance dose of 5 mg daily, with an INR of 2.72 at discharge. The patient was discharged home on day 8 with only mild residual left central facial palsy and subtle weakness of the left upper limb. At the 3-month follow-up, she had achieved an excellent functional outcome, with a modified Rankin Scale (mRS) score of 0.

## Discussion

In this report, we describe the administration of IVT for acute cerebral infarction following the urgent reversal of warfarin’s anticoagulant effect with 4F-PCC in two patients with mechanical aortic valve replacements. In both cases, this approach was not associated with hemorrhagic complications, including SICH, and both patients achieved a favorable functional outcome. The use of 4F-PCC is an effective method for achieving an immediate reduction and normalization of the INR, particularly in urgent situations such as severe or life-threatening bleeding or the need for rapid reversal of anticoagulation before an acute surgical or interventional procedure. Although treatment with 4F-PCC carries a risk of prothrombotic and thromboembolic complications, a meta-analysis of clinical trials indicates that this risk is relatively low, not exceeding 2.5% (13).

To date, the published evidence regarding the use of 4F-PCC for reversal of VKA-induced anticoagulation before IVT is limited to several case reports and a single case series (14–18). Chausson et al. reported the largest series, including 26 patients with acute ischemic stroke who were successfully treated with this approach in combination with intravenous vitamin K administration (15). The mean baseline INR was 2.3 ± 0.6 and decreased to 1.3 ± 0.2 within 5 minutes after anticoagulation reversal. No patient developed symptomatic intracranial hemorrhage or an acute thrombotic complication. One patient experienced hemorrhoidal bleeding requiring blood transfusion. At 3 months, 61.5% of patients had achieved functional independence (modified Rankin Scale [mRS] ≤ 2).

INR was measured immediately after the administration of 4F-PCC in both patients. In the first patient, the INR decreased from 3.2 to 1.2, and in the second patient, from 1.91 to 1.3. In both cases, IVT was performed only after confirming effective reversal of the anticoagulant effect of warfarin, specifically at an INR of ≤1.7. During regular monitoring within the first 24 h following IVT, the INR remained below 1.7 in both patients, specifically within the range of 1.4–1.7. Vitamin K was not administered concurrently in either case.

This approach cannot be considered a standard strategy for reversing the effects of VKAs, as current practice generally recommends a combination of 4F-PCC and vitamin K. However, in our cases, the decision was influenced by the specific clinical situation of patients with mechanical aortic valve replacements. While the effect of 4F-PCC is almost immediate, vitamin K has a delayed onset of action and provides more sustained reversal of the anticoagulant effect. This difference is explained by the mechanisms of action of these agents: 4F-PCC allows for the rapid replenishment of vitamin K-dependent coagulation factors, whereas vitamin K restores the endogenous synthesis of these factors; consequently, its effect has a delayed onset but persists longer. 4F-PCC was selected to achieve rapid INR correction, with an effect occurring within minutes, rather than fresh frozen plasma, the effect of which may take several hours to manifest.

In patients with mechanical heart valves, this prolonged interruption of anticoagulation may increase the risk of prosthetic valve thrombosis, a rare but potentially life-threatening complication. Given the concurrent administration of IVT, our aim was to achieve rapid, laboratory-confirmed reversal of warfarin’s effect while minimizing the duration of anticoagulation interruption. Administration of vitamin K would have resulted in a delayed recovery of coagulation function and could have prolonged the period during which the mechanical valve was without effective anticoagulant protection. We considered this particularly important given the need to resume anticoagulant therapy as soon as possible.

Our decision therefore represented an individualized risk–benefit assessment based on the specific clinical situation and should not be interpreted as a general recommendation to omit vitamin K when reversing the effects of warfarin. The period following IVT administration also represents a critical phase from two perspectives. The use of anticoagulant or antiplatelet therapy is generally contraindicated during the first 24 h after IVT administration because of the risk of hemorrhagic complications. Conversely, the absence of adequate anticoagulation carries a risk of thrombotic complications involving the mechanical valve. We therefore considered the safest approach to be performing a follow-up CT scan after a shorter interval (18 h after IVT in the first case and 19 h in the second) and, after ruling out intracranial hemorrhagic complications, immediately initiating anticoagulation with LMWH. In both patients, full anticoagulant therapy with warfarin was resumed concurrently following the follow-up CT scan. The therapeutic INR range, and thus the possibility of discontinuing LMWH therapy, was achieved on the fourth day after initiation of warfarin.

In both cases, 4F-PCC, which contains coagulation factors II, VII, IX, and X, was used. The choice of 4F-PCC over 3F-PCC was based on the need for rapid and predictable correction of warfarin-induced coagulopathy. While 3F-PCC contains factors II, IX, and X, 4F-PCC also contains factor VII; replenishment of this factor contributes to the rapid normalization of prothrombin time and INR. Available comparative studies and meta-analyses indicate that 4F-PCC achieves target INR correction more frequently than 3F-PCC, while no clear difference in the risk of thromboembolic complications has been demonstrated (19). We therefore regarded the presence of factor VII in 4F-PCC not as the addition of another nonspecific procoagulant factor, but rather as an integral component of the adequate replacement of vitamin K-dependent coagulation factors suppressed by warfarin.

Several significant limitations must be highlighted when interpreting our report. First, the findings are based on only two case reports, which cannot provide a sufficient basis for evaluating the safety or efficacy of this therapeutic approach or for modifying current guidelines. The favorable clinical course observed in both patients cannot be generalized to the broader population of patients with acute ischemic stroke treated with warfarin, and the potential selection bias inherent in individual clinical decision-making cannot be ruled out. Therefore, the safety and efficacy of IVT following VKA reversal with 4F-PCC should be evaluated in larger observational cohorts and, subsequently, if feasible, in prospective studies.

When considering this approach for an individual patient, other risk factors for intracranial and systemic hemorrhage must be assessed. In addition to the current INR value, particular consideration should be given to a history of severe bleeding, prior ICH, uncontrolled arterial hypertension, and the extent of early ischemic changes. The presence of cerebral microbleeds also warrants attention; although they are associated with an increased risk of ICH, current guidelines do not consider them an absolute contraindication to IVT (10, 12). Given that brain MRI performed during the acute phase of ischemic stroke can significantly prolong the time to initiation of IVT, the potential benefit of this examination must always be weighed against the patient’s clinical status and the available therapeutic time window.

A patient’s ethnic background may also be a potentially relevant factor. Epidemiological data indicate differences in the incidence and distribution of stroke subtypes across ethnic populations; notably, a higher incidence of intracerebral hemorrhage has been reported in certain Asian populations compared with populations of European descent (20). However, these differences cannot be directly extrapolated to an individual’s risk of symptomatic intracranial hemorrhage following IVT. Data on the combination of warfarin reversal with 4-factor prothrombin complex concentrate (4F-PCC) followed by IVT are very limited. Therefore, ethnicity should be considered as one factor in the overall risk assessment rather than as a standalone criterion for indicating or contraindicating treatment. According to a recent meta-analysis, no significant differences were observed between Asian and White populations regarding the incidence of symptomatic intracranial hemorrhage (*p* = 0.20) or any intracranial hemorrhage (*p* = 0.83) based on the choice of thrombolytic agent (alteplase versus tenecteplase) Similarly, among patients treated with tenecteplase, no significant differences in sICH rates were found across subgroups of Asian, White, and mixed ethnic origin (*p* = 0.33) (21).

In the cases presented here, rapid normalization of prothrombin time and stabilization of coagulation occurred shortly after the administration of 4F-PCC, as evidenced by INR values of 1.2 and 1.3 prior to the initiation of IVT. However, the favorable outcomes observed in both cases cannot be interpreted as evidence of the safety of this approach. These are two isolated case reports without a control group and without the ability to determine the incidence of hemorrhagic or thromboembolic complications. Consequently, the results cannot be generalized to other patients with mechanical heart valves or to patients with different risk profiles.

In conclusion, the two cases presented here demonstrate that urgent reversal of the anticoagulant effect of warfarin using 4F-PCC resulted in rapid normalization of the INR, followed by IVT without subsequent development of SICH or other serious complications. However, given the small number of cases, these observations do not provide a sufficient basis for evaluating the safety or efficacy of this approach or for modifying current guidelines. In patients with a mechanical heart valve and acute ischemic stroke presenting with an INR > 1.7, reversal of warfarin’s effect with 4F-PCC followed by IVT may represent a potential therapeutic option in selected, individually assessed cases, provided that ischemic and hemorrhagic risks are carefully evaluated, including the patient’s history of bleeding complications and available clinical and radiological risk factors. Further clinical data are needed to assess the safety and efficacy of this approach.

## Data Availability

The original contributions presented in the study are included in the article/supplementary material, further inquiries can be directed to the corresponding author.
